# Constitutive gp130 activation rapidly accelerates the transformation of human hepatocytes via an impaired oxidative stress response

**DOI:** 10.18632/oncotarget.10956

**Published:** 2016-07-30

**Authors:** Denise Heim, Ines Gil-Ibanez, Johannes Herden, Ann Christin Parplys, Kerstin Borgmann, Dirk Schmidt-Arras, Ansgar W. Lohse, Stefan Rose-John, Henning Wege

**Affiliations:** ^1^ Department of Gastroenterology and Hepatology, University Medical Center Hamburg-Eppendorf, 20246 Hamburg, Germany; ^2^ Laboratory of Radiobiology and Experimental Radiooncology, University Medical Center Hamburg-Eppendorf, 20246 Hamburg, Germany; ^3^ Institute of Biochemistry, Christian-Albrechts-Universität zu Kiel, 24098 Kiel, Germany

**Keywords:** interleukin 6, glycoprotein 130, reactive oxygen species, oxidative stress response, hepatocyte transformation

## Abstract

Pro-inflammatory signaling pathways, especially interleukin 6 (IL-6), and reactive oxygen species (ROS) promote carcinogenesis in the liver. In order to elucidate the underlying oncogenic mechanism, we activated the IL-6 signal transducer glycoprotein 130 (gp130) via stable expression of a constitutively active gp130 construct (L-gp130) in untransformed telomerase-immortalized human fetal hepatocytes (FH-hTERT). As known from hepatocellular adenomas, forced gp130 activation alone was not sufficient to induce malignant transformation. However, additional challenge of FH-hTERT L-gp130 clones with oxidative stress resulted in 2- to 3-fold higher ROS levels and up to 6-fold more DNA-double strand breaks (DSB). Despite increased DNA damage, ROS-challenged FH-hTERT L-gp130 clones displayed an enhanced proliferation and rapidly developed colony growth capabilities in soft agar. As driving gp130-mediated oncogenic mechanism, we detected a decreased expression of antioxidant genes, in particular glutathione peroxidase 3 and apolipoprotein E, and an absence of *P21* upregulation following ROS-conferred induction of DSB. In summary, an impaired oxidative stress response in hepatocytes with gp130 gain-of-function mutations, as detected in dysplastic intrahepatic nodules and hepatocellular adenomas, is one of the central oncogenic mechanisms in chronic liver inflammation.

## INTRODUCTION

Among all cancers, hepatocellular carcinoma (HCC) is the most prevalent inflammation-associated human cancer with more than 90% of all cases arising in the context of chronic hepatic injury and inflammation. Interestingly, inflammatory infiltrates and overexpression of acute phase inflammatory response genes at the molecular level are also present in inflammatory hepatocellular adenomas (IHCA), benign liver tumors with risk for malignant transformation [1, 2]. Therefore, a functional understanding of pro-inflammatory signaling pathways and inflammation-associated cellular mechanisms involved in the transition from adenomatous liver lesions or chronic liver injury to hepatocyte transformation will be crucial to develop new therapeutic approaches.

Interleukin 6 (IL-6) is one of the best-characterized pro-tumorigenic cytokines. It signals through several well-described pathways, in particular the Janus kinase (JAK)-signal transducer and activator of transcription 3 (STAT3) pathway, the Src homology 2 (SH2)-containing protein tyrosine phosphatase-2 (SHP-2)-Ras-Raf-MEK-extracellular signal-regulated kinase (ERK) pathway and the phosphoinositide-3-kinase (PI3K)-Akt pathway [3]. Activation of the downstream signaling target STAT3 has been identified in several human malignancies, including myeloma, prostate cancer, melanoma, ovarian cancer, renal cell carcinoma, and breast cancer [4]. As leading mechanism for STAT3 activation in inflammation-associated tumors, activation of the signal transducer gp130 via the pro-inflammatory cytokine IL-6 has been discussed [5]. To this regard, IL-6 has been identified as an independent risk factor for tumor development in patients with chronic hepatitis B [6, 7]. In the case of premalignant adenomatous liver lesions, somatic gain-of-function mutations in the gp130 gene (*IL6ST*) were found in 60% of analyzed IHCA [8]. The detected mutations resulted in ligand-independent activation of gp130 and constitutive STAT3 phosphorylation. In HCC, gp130 mutations always occur together with β-catenin-activating mutations, suggesting a cooperative effect in the malignant transformation of hepatocytes [2]. However, the functional role of ligand-independent IL-6 signaling in the transformation process of hepatocytes (e.g., in the transition from IHCA to HCC) and the underlying cellular mechanisms are not entirely understood. Besides inflammatory cytokines, oxidative stress, characterized by the generation of reactive oxygen species (ROS), also plays a central role in inflammation-mediated transformation. For example, accelerated hepatocarcinogenesis following liver resection in *Mdr2^−/−^* mice, a model for inflammation-associated liver cancer, was associated with increased ROS levels and a higher frequency of DNA-double strand breaks (DSB) [9].

In this study we utilized untransformed proliferating telomerase-immortalized human fetal hepatocytes (FH-hTERT), as the currently only available human hepatocyte cell culture model, to explore the cellular mechanisms promoting ROS-induced transformation in the context of activated IL-6 signaling.

## RESULTS

### Activation of gp130-signaling

**Figure 1:**
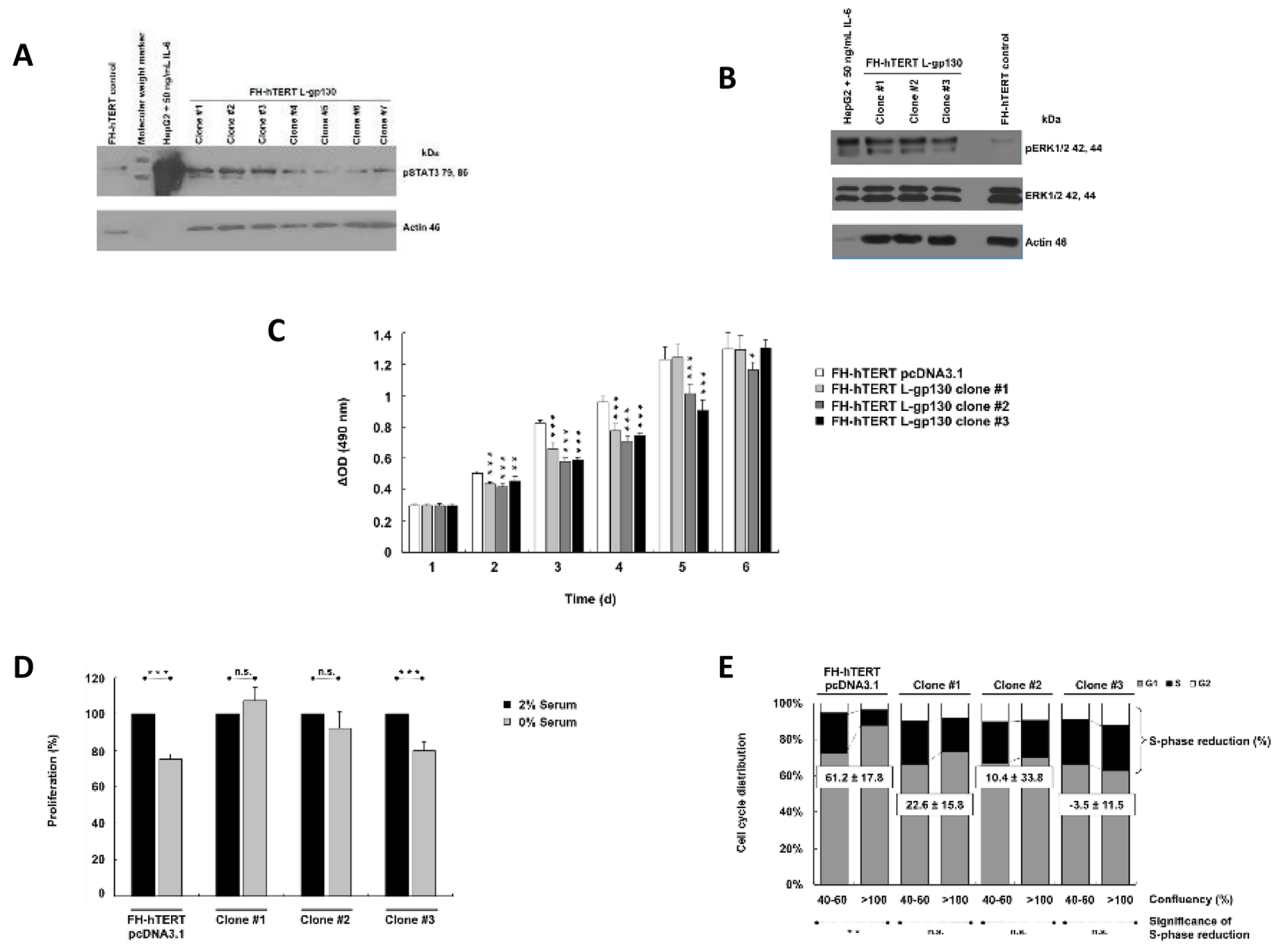
Constitutive activation of IL-6 signaling and phenotype characterization **A.** Phosphorylation of STAT3 and **B.** ERK1/2 were evaluated by immunoblotting with up to 40 μg protein per lane. HepG2 cells (5 μg per lane) treated with 50 ng/ml IL-6 for 10 min served as positive control. β-Actin and ERK1/2 were visualized as loading control. **C.** Cell proliferation was monitored by colorimetric measurement of cell density at different time points after seeding. Depicted are means ± SD (error bars). **D.** To investigate serum dependence, cells were cultured with 0 or 2% serum. Cell density was determined at day 3 after seeding by colorimetric assay. Bar graphs represent the average cell proliferation ± SD (error bars) of six wells relative to 2% serum (= 100%). **E.** Cell cycle profiles of near-confluent (40-60%) and super-confluent (>100%) cultures were obtained by flow cytometry. Histograms were analyzed by FlowJo to attain the different fractions of the cell cycle. Bar graphs show cell cycle distribution and calculated S-phase reduction (in % of super-confluent cultures; negative S-phase reduction indicates an increase). P-values are as indicated, *P* ≤0.05 (*), *P* ≤0.01 (**), *P* ≤0.001 (***), and not significant (n.s.).

In this study, we mimicked permanent IL-6 signaling by stable transfection of FH-hTERT with a constitutively active signal transducer gp130 construct (L-gp130). Following antibiotic selection, seven single cell-clones were isolated and expanded. To verify robust gp130-mediated signal transduction in these clones, we analyzed phosphorylation of STAT3. In comparison to FH-hTERT transfected with the empty vector pcDNA3.1 (in the following labeled as mock-transfected), three of the seven clones (L-gp130 clone #1, #2, and #3) showed an increased band intensity for pSTAT3 (Figure 1A). Additionally, we evaluated pERK1/2 and detected an increased band intensity in the three clones (Figure 1B).

### Phenotype characterization of L-gp130 clones

To characterize functional consequences induced by the activation of gp130-mediated signaling, we monitored cellular growth properties. Contrary to our assumption, L-gp130 clones did not show an accelerated cellular growth in comparison to mock-transfected FH-hTERT (Figure 1C). The clones even displayed a decelerated growth pattern. Serum-free culture conditions significantly reduced cellular growth in the control cells and clone #3, with a 20-30% reduction in cell density compared to cells cultured with 2% serum (Figure 1D). However, we detected no significant growth reduction for clone #1 and #2, and therefore, serum dependence was abolished in these clones. To detect changes in contact inhibition, we compared S-phase fractions in confluent cultures with log-proliferating cultures. In mock-transfected cells, the S-phase fraction was reduced by more than 60% in confluent cultures, confirming effective growth inhibition by cell-to-cell contacts. However, in L-gp130 clone #1 and #2, contact inhibition was strongly reduced, and in clone #3 completely eliminated with an increased S-phase fraction (Figure 1E). The divergent growth response following gp130 activation may be due to the magnitude of pathway activation in each L-gp130 clone. To investigate anchorage-independent growth, an established *in vitro* marker for a malignant phenotype, we scored colony formation in soft agar. The results are listed in Table 1. Immediately after stable transfection and selection (passage 8), no colony formation was observed in L-gp130 clones #1 to #3. Interestingly, all three clones expanded in long-term culture developed the ability to generate colonies in soft agar beyond approximately passage 60-65. In contrast, no colony growth was scored for mock-transfected FH-hTERT. Corresponding to the colony assay data, tumor formation was observed in athymic nude mice transplanted with L-gp130 clones (passage 8) after a latency period of 2-3 months. Tumors formed in 67% of clone #1 and in 33% of clone #3 transplantation sites. As expected, no tumor formation was detected in mice transplanted with mock-transfected FH-hTERT during a 1-year observation period (Table 2).

**Table 1:** Anchorage-independent growth

Cell clone	Passage 8		Passage >60-65		
	Cells seeded	15,000	5,000	10,000	20,000
FH-hTERT control		0	0	1 ± 1	0
Clone #1		0	16 ± 4	45 ± 7	118 ± 18
Clone #2		0	28 ± 9	67 ± 8	143 ± 9
Clone #3		0	27 ± 6	63 ± 7	137 ± 11

Mean ± SD.

**Table 2:** Tumor formation in athymic nude mice

Clone	Sites	Tumors (%)	Weeks
FH-hTERT control	6	0	NA
Clone #1	6	4 (66.7)	8.5 ± 0.7
Clone #2	6	0	NA
Clone #3	6	2 (33.3)	10.3 ± 0

NA, not applicable. Mean ± SD.

### Challenge with oxidative stress

**Figure 2:**
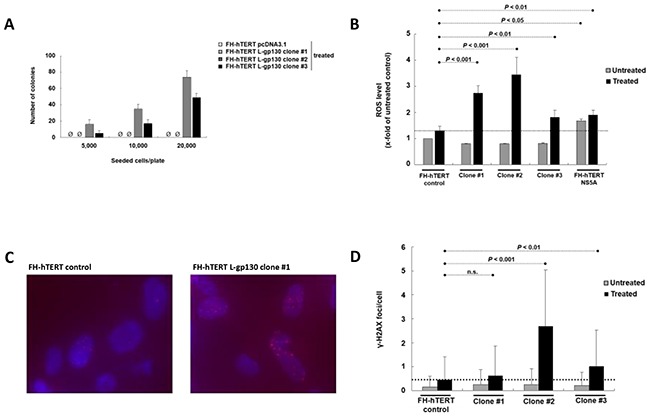
Challenge with oxidative stress and consequences of higher ROS levels **A.** Colony formation induced by oxidative stress. Number of colonies per 5000, 10,000, and 20,000 seeded cells on 60-mm tissue culture dishes after 4 weeks. Treatment with H2O2/BSO was performed 12 h before seeding. **B.** Levels of ROS with and without induction of oxidative stress. ROS levels of untreated cells (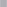) are compared to ROS levels after H2O2/BSO treatment for 90 min (■). In comparison to treated mock-transfected FH-hTERT (dashed line), H2O2/BSO treatment resulted in higher ROS levels in L-gp130 clones. FH-hTERT NS5A served as an additional positive control. **C.** γ-H2AX foci were visualized by fluorescent staining. The photographs show representative pictures of L-gp130-positive cells and FH-hTERT control after challenge with ROS. **D.** Quantitative analysis of γ-H2AX foci formation expressed as average amount of foci per cell ± SD (error bars).

**Figure 3:**
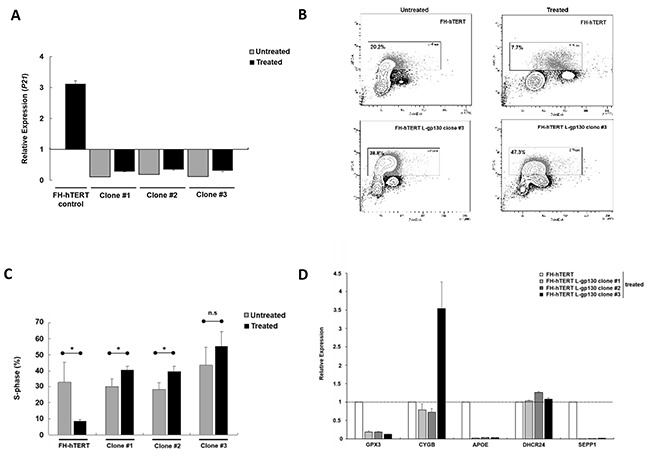
Mechanisms of ROS-induced transformation **A.** Levels of P21. RNA was extracted after 8 h treatment and relative expression levels were determined by qPCR with a basket housekeeper as internal control and mock-transfected FH-hTERT as calibrator (expression level = 1). Bar graphs represent average expression levels ± SD (error bars). **B.** Flow cytometric measurement of total DNA and incorporated BrdU. Representative BrdU APC-A vs. DNA 7-AAD-A plots (FSC-A vs. SSC-A gated cell population) showing G0/G1, S, and G2/M fractions from treated and untreated control and L-gp130-positive cells. Cells in S-phase are framed. **C.** Cell proliferation under oxidative stress determined by BrdU incorporation assay. Depicted are average S-phase fractions ± SD (error bars) of treated (■) and untreated (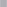) clones and control cells. P-values are as indicated, *P* ≤0.05 (*), *P* ≤0.01 (**), *P* ≤0.001 (***), and not significant (n.s.). **D.** Changes in gene expression post ROS exposure. Relative expression of GPX3, CYGB, APOE, DHCR24, and SEPP1 in L-gp130 clones in comparison to FH-hTERT control (expression level=1).

In chronic hepatitis, gene alterations are driven by ROS. To elucidate downstream mechanisms driving the transformation process, we challenged our L-gp130 clone #1 to #3 in early expansion culture with oxidative stress and monitored phenotype changes by soft agar assay. Challenge with oxidative stress accelerated the transformation process occurring in long-term culture (see above) and colony formation was detected rapidly after treatment with hydrogen peroxide/DL-buthionine-[S,R]-sulfoximine (H_2_O_2_/BSO) (Figure 2A). In order to dissect the underlying gp130- and ROS-induced mechanisms, we measured ROS levels. As expected, FH-hTERT expressing the non-structural hepatitis C virus protein NS5A showed higher ROS levels, even without additional ROS challenge (Figure 2B). In contrast, our untreated L-gp130 clones showed ROS levels comparable to untreated mock-transfected FH-hTERT. Remarkably, challenge with H_2_O_2_/BSO resulted in up to 3-fold higher ROS levels in our clones #1 to #3 compared to treated control cells (Figure 2B). To test if these higher ROS levels result in more DNA damage, we visualized DSB by immunofluorescent staining for γ-H2AX (Figure 2C). Without ROS challenge, clone #1 to #3 with active gp130 showed similar quantities of γ-H2AX foci in comparison to mock-transfected cells (Figure 2D). After H_2_O_2_/BSO treatment, however, we observed an up to 6-fold increase in the number of γ-H2AX foci in our L-gp130 clones. Next, we monitored cell cycle arrest after induction of oxidative stress by testing the expression of *P21*, which is a transcriptional target of p53 and plays a crucial role in mediating cell cycle arrest in cells with DSB. Compared to untreated control cells, untreated L-gp130 clones showed lower expression of the regulator *P21*. However, similar to the control cells *P21* expression was upregulated in response to higher ROS levels in all three L-gp130 clones. Moreover, although H_2_O_2_/BSO treated clones displayed significantly higher ROS levels resulting in more DSB (see above), expression of *P21* was lower compared to treated mock-transfected FH-hTERT (Figure 3A). Determination of S-phase fractions by BrdU-incorporation of treated and untreated cells confirmed the lack of *P21*-mediated cell cycle arrest. Representative plots are shown in Figure 3B. Without treatment, S-phase fractions of all L-gp130 clones were between 30-40% (Figure 3C). Following H_2_O_2_/BSO treatment, FH-hTERT control cells displayed a reduction in the S-phase fraction compared to untreated control cells, which indicates robust cell cycle arrest induced by oxidative stress. In sharp contrast, our L-gp130 clones did not show a reduced S-phase fraction, but rather exhibited an enhanced proliferation following H_2_O_2_/BSO treatment.

### Antioxidative stress response

In order to understand the higher ROS levels in our clones following H_2_O_2_/BSO treatment, we profiled the expression of various genes related to oxidative stress and the response mechanisms employing PCR array technology. The assay revealed an upregulation of glutathione peroxidase 3 *(GPX3)* and cytoglobin (*CYGB*) in all three L-gp130 clones in comparison to mock-transfected cells (Supplementary Figures S1A and S1C). Expression of the antioxidant apolipoprotein E *(APOE)* was 10-fold downregulated (Supplementary Figure S1D), and *2*4-dehydrocholesterol reductase (*DHCR24*) and selenoprotein P (*SEPP1*), oxidative stress responsive genes, were also considerably altered in the three clones (Supplementary Figure S1H). Relevant genes detected by this PCR array, were further monitored by quantitative real-time PCR (qPCR) in our L-gp130 clones with and without additional ROS challenge (Figure 3D). Despite H_2_O_2_/BSO treatment, all three L-gp130 clones showed lower expression levels for *GPX3*, *APOE*, and *SEPP1*, compared to treated FH-hTERT control cells. In addition, expression of *CYGB* was much higher in clone #3 than in the control cells. Expression levels of *DHCR24* were similar in all treated cells. Furthermore, we measured the expression of *NQO1*, coding for NAD(P)H dehydrogenase (quinone) 1. *NQO1* is a transcriptional target of the master antioxidant regulator nuclear factor (erythroid-derived 2)-like 2 (NRF2). We quantified the expression of *NQO1* in all three L-gp130 clones without ROS-challenge. Two clones showed slightly higher *NQO1* expression in comparison to untreated FH-hTERT control cells (Supplementary Figure S2); however, we could not detect a uniform and reproducible alteration in *NQO1* expression (as marker for NRF2 activation) in our gp130-activated clones.

## DISCUSSION

Under normal conditions, activation of STAT3 (downstream of gp130) induces cyclin D1, D2, and B1, as well as c-myc, and downregulates the expression of *P21*, thus promoting entry into the cell cycle [10]. Furthermore, IL-6 (upstream of gp130) is essential to control cell proliferation in healthy hepatocytes [11]. However, and in contrast to these findings, proliferation was not enhanced in our immortalized hepatocyte clones FH-hTERT following constitutive gp130 activation, although STAT3 was activated and *P21* expression was lower than expected. In line with our observation, Santer *et al.* demonstrated an anti-proliferative effect of IL-6 in prostate cancer cell lines [12]. Thus, our data indicate that the growth-promoting effect of gp130 may be restricted to otherwise quiescent cells, e.g. hepatocytes in liver inflammation. In our study, constitutive gp130 activation induced a less serum-sensitive cell growth, and decreased contact inhibition. Interestingly, colony formation in soft agar, an established *in vitro* indicator for malignant transformation, was not present after transfection with L-gp130, but developed in all three clones during long-term expansion culture. Corresponding to the colony assay data, tumors formed in mice transplanted with Lgp-130 clones. In summary, all three analyzed L-gp130 clones displayed a pre-malignant phenotype; however, a transformed phenotype (colony formation) was not observed immediately after transfection with gp130 construct. These findings confirm the feasibility of our model for the investigation of gp130- and ROS-associated mechanisms promoting the transformation processes in proliferating hepatocytes. Furthermore and in accordance with our findings, transgenic animals with overexpression of *IL-6* and *IL-6R* under the control of a liver-specific promoter develop nodules of hepatocellular hyperplasia identical to human nodular hyperplasia of an inflammatory background [13]. Over the time, these lesions progress into large liver adenomas, but not HCC. Regarding human HCC, tumors with gp130 mutations always display an additional activating mutation of the β-catenin pathway, indicating that additional hits are required for malignant transformation of hepatocytes with forced gp130 activation [8].

Alterations in redox balance and oxidative stress levels are a hallmark of carcinogenesis [14]. ROS activate various signaling cascades that regulate cell growth and transformation [15]. Furthermore, oxidative stress causes DNA damage by inducing the formation of DNA adducts, such as 8-oxo-2′-deoxyguanosine [16]. Along this line, patients with chronic hepatitis showed an increased production of ROS and higher levels of 8-oxo-2′-deoxyguanosine in hepatocytes with high risk for transformation [17]. Moreover, elevated oxidative stress was reported in the *Mdr2^−/−^* mice in the chronic inflammatory stages, indicating that oxidative stress plays a pivotal driving role in hepatocyte transformation [18]. To investigate the oncogenic potential of oxidative stress in human hepatocytes with activated IL-6 signaling, we induced ROS by treating our clones with H_2_O_2_/BSO. Challenge with oxidative stress strikingly accelerated the transformation process observed in long-term culture and colony formation was detected rapidly after H_2_O_2_/BSO treatment. Furthermore, after challenge with oxidative stress, ROS levels were up to 3-fold higher in L-gp130 clones compared to treated mock-transfected FH-hTERT (control cells without gp130 activation). These higher ROS levels were associated with an increased frequency of DNA damage, as demonstrated by staining for DSB. In general, DSB activate the DNA damage response machinery that in turn leads to cell cycle arrest, DNA repair, and apoptosis. To examine DSB-induced cell cycle control mechanisms in our L-gp130 clones, *P21* expression was measured by qPCR and S-phase fractions were determined by the measurement of BrdU incorporation. Surprisingly, L-gp130 clones did not enter cell cycle arrest despite higher ROS and DSB levels and even displayed an enhanced proliferation. Fuke and colleagues have shown that inhibition of JAK/STAT3 signaling in HepG2 cells (with constitutive STAT3 activation) exerts an antiproliferative effect on the cell-cycle while p16 and p21 are upregulated [19]. Moreover, Phesse and co-workers have observed that partial suppression of JAK/STAT3 signaling in APC-mutant mice is sufficient to diminish tumor growth by reducing Bmi-1 dependent repression of p21 and p16 [20]. Based on our data and these reports, impairment of p21 activation in our cell clones may directly be triggered by gp130 overexpression. However, further investigations are required to fully characterize this interaction. Nevertheless, our findings corroborate the key observations regarding the role of IL-6 in chronic liver inflammation and hepatocarcinogenesis in murine liver cancer models. For example, in the *Mdr2^−/−^* model, hepatocytes with genomic instability are much more likely to escape from cell cycle arrest following partial hepatectomy with consecutive IL-6-mediated regenerative stress [9]. In our cell clones, decreased cell cycle arrest due to gp130 activation promotes the accumulation of unrepaired DSB, thus facilitating transformation.

To identify the gp130-related mechanism leading to higher ROS levels in our clones, we profiled the expression of various genes related to oxidative stress and the associated response mechanisms. Despite similar ROS levels without treatment, we detected a significantly altered expression of *GPX3*, *CYGB*, *APOE*, *DHCR24*, and *SEPP1* in our L-gp130 clones. GPX3 belongs to the family of glutathione peroxidases, the most important ROS scavengers [21], and its expression is suppressed in a variety of cancers. A strong suppression is reported for prostate cancer, thyroid cancer, colorectal cancer, gastric cancer, and breast cancer [22, 23]. Interestingly, *GPX3* expression was higher in clear cell epithelial ovarian carcinoma tissue compared to healthy control, suggesting a tumor-specific activity of GPX3 [24]. The expression of *GPX3* in the transformation of hepatocytes has not been studied so far. APOE is a glycoprotein and a ligand for the LDL receptor or the remnant receptor involved in lipid metabolism [25]. Yokoyama *et al.* reported increased APOE protein levels in 88% of HCC patients, but without upregulation in *APOE* gene expression and serum levels, thus suggesting an accumulation by impaired secretion [26]. In our gp130-activated clones, a robust decrease in *APOE* expression was observed after H_2_O_2_/BSO treatment. SEPP1 acts as a selenium transport protein, a heavy-metal chelator, and an antioxidant [27]. In normal mucosa, *SEPP1* is highly expressed whereas a significant reduction or loss of *SEPP1* expression is detected, for instance, in colon cancer [28]. *SEPP1* is also suppressed in a subset of human prostate tumors, mouse tumors, and several prostate cancer cell lines [29]. *CYGB* expression was slightly downregulated in two out of three L-gp130 clones after challenge with oxidative stress. *CYGB* overexpression protects the human neuronal cell line TE671 from pro-oxidant RO19-8022-induced DNA damage [30]. Moreover, *in vitro* and *in vivo* overexpression of *CYGB* in rat hepatic stellate cells protects these cells against oxidative stress [31]. A downregulation has been reported in several human cancers, in particular head and neck cancer, ovarian cancer, and breast cancer [32]. Furthermore, loss of *CYGB* in *Cygb^−/−^* mice increased their susceptibility to diethylnitrosamine-induced tumorgenesis, indicating a tumor suppressive function of CYGB [32]. High levels of the oxidoreductase DHCR24 mediate resistance against oxidative stress and prevent apoptotic cell death [33]. Induction of *DHCR24* by hepatitis C virus also impairs apoptosis induced by oxidative stress and inhibits p53 [34]. In our immortalized clones, we observed a slightly increased *DHCR24* expression after ROS treatment as a result of gp130 activation. Additionally, we measured *NQO1* expression, one of the target genes of NRF2, regarded as the principal regulator of cytoprotective and antioxidant genes. Recently, a meta-analysis has suggested that *NQO1* variant alleles and genotypes are significantly related to an increased risk of hepatocarcinogenesis [35]. In our cell clones, we could not detect a universal and reproducible alteration of NQO1 expression as a consequence of gp130 activation. Therefore, these data do not suggest a direct interaction between gp130 and NRF2.

In summary, our data demonstrate that permanent genetically driven IL-6 pathway activation (by gain-of-function mutations in *gp130*), directly impairs oxidative stress response, causing higher ROS levels and subsequently a higher frequency of DSB. In addition, enhanced cell cycle turnover driven by gp130 pushes pre-malignant cells with genetic alterations through additional cell cycle rounds and leads to the accumulation of unrepaired DSB, finally resulting in the transformation of our L-gp130 clones (and cells in IHCA). This finding explains the rather high frequency of *gp130* mutations in HCC that have developed from IHCA or dysplastic intrahepatic nodules in chronic liver inflammation.

## MATERIALS AND METHODS

### Cell lines

Immortalized FH-hTERT cells were used at population doubling 35-40 [36]. HepG2 cells (obtained from ATCC, HB-8065) were cultured as control human HCC cell line. FH-hTERT stably transfected with the hepatitis C virus non-structural protein NS5A served as an additional control.

### Plasmids and transfection

To mimic activated IL-6 signaling, FH-hTERT were stably transfected with the constitutively active L-gp130 construct [37]. The empty vector pcDNA3.1 (Invitrogen, Karlsruhe, Germany) was transfected as vector control. Cells were nucleofected using Nucleofector Solution V (Amaxa, Köln, Germany) and program T30, and subsequently selected with 200 μg/ml Zeocin (Invitrogen) for L-gp130 and 200 μg/ml Geneticin (Invitrogen) for the empty vector control.

### Selection of single-cell clones

We derived single cell-clones with stable expression of L-gp130 by seeding 100 cells of the transfected culture on 60-mm tissue culture dishes. During the following 2-3 weeks, seven single-cell clones were picked with cloning rings (Sigma-Aldrich, Seelze, Germany) and expanded for further characterization.

### Immunoblot to determine downstream pathway activation

Proteins were extracted and quantified with the BCA Protein Assay Kit (Pierce Biotechnology, Rockford, IL, USA). Primary antibody solution detecting pSTAT3 (1:1000; #9131; Cell Signaling Technology, Danvers, MA, USA) or pERK1/2 (1:1000; #9101; Cell Signaling Technology) were incubated at 4°C overnight. Incubation with the secondary antibody solution conjugated with horseradish peroxidase was performed for 1 h at room temperature. Visualization was achieved by enhanced chemiluminescence. Actin (1:2000; #sc-1616; Santa Cruz Biotechnology, Santa Cruz, CA, USA), STAT3 (1:1000; #9132; Cell Signaling Technology), and ERK1/2 (1:1000; #4695; Cell Signaling Technology) served as loading controls.

### Cell proliferation and serum dependence

Proliferation was monitored as described [38]. To investigate serum dependence, cells were seeded and serum was omitted or changed to 2% in the following medium changes.

### Contact inhibition

Cell-cycle profiles of near-confluent (40-60%) and super-confluent cultures (culture for >1 day after reaching 100% confluency) were obtained by FACS evaluation as described [39], employing the operator-independent analysis software FlowJo 7.5 (FlowJo, LLC; Ashland, OR, USA).

### Anchorage-independent growth

Soft agar colony assays were performed as previously summarized [36].

### Tumor formation in nude mice

To investigate malignant transformation *in vivo*, L-gp130 clones were transplanted in approximately 10-week-old athymic nude mice (NMRI-*Foxn1^nu^*, female, 21-25 g body weight; Charles River Laboratories, Sulzfeld, Germany). Animal protocols were approved by the local review board (protocol number 6/12) and the experiments were performed as described before [38].

### Induction and quantification of oxidative stress

To induce oxidative stress, we treated the cells with 400 μM H_2_O_2_ and 5 μM BSO, a glutathione depleting agent (Sigma-Aldrich). If not mentioned otherwise, treatment was completed after 12 h. ROS levels were measured by FACS analysis using carboxy-H_2_DCFDA (Invitrogen). In brief, 0.25 × 10^6^ cells were seeded in tissue culture flasks (T25, 25 cm^2^) and cultured until 80-90% confluency and washed with HBSS. After incubation with 25 μM carboxy-H_2_DCFDA diluted in HBSS for 30 min at 37°C, ROS treatment was performed for 60-90 min.

### Detection of DNA double strand breaks

To quantify DSB after H_2_O_2_/BSO treatment, we visualized γH2AX foci by fluorescent staining according to Koch *et al.* [40]. For quantitative analysis, foci were counted with a Zeiss Axioplan 2 imaging microscope (Zeiss, Oberkochen, Germany) using a 63-fold magnification. One hundred cells per slide and experiment were evaluated blindly.

### Incorporation of BrdU

To detect the effect of oxidative stress on DNA synthesis, proliferation was determined with the APS BrdU Flow Kit (BD Biosciences, Heidelberg, Germany). Cells were plated at a density of 0.35 × 10^6^ cells per T25 and cultured until 70-80% confluency. Following the indicated treatment, cells were incubated for 45 min at 37°C with BrdU at a final concentration of 10 μM in cell culture medium. Fixation and staining was performed according to the manufacturer's protocol. Finally, S-phase fractions were determined using a FACSCanto flow cytometer (BD Biosciences).

### Gene expression analysis

RNA was extracted with NucleoSpin RNA II Kit (Machery-Nagel, Bethlehem, PA, USA) and reverse transcribed with Transcriptor First Strand cDNA Synthesis Kit (Roche Diagnostics, Penzberg, Germany). After diluting the cDNA synthesis reaction with ddH_2_O (1:5.55), expression profiling was executed with the Human Oxidative Stress and Antioxidant Defense RT^2^ Profiler PCR Array (SABiosciences, Frederick, MD, USA) on an ABI Prism 7900H thermal cycler (Applied Biosystems, CA, USA). Expression levels were determined by comparative quantification (2^−ΔΔCt^) employing mock-transfected FH-hTERT as calibrator (expression level = 1). Results were confirmed by qPCR for *GPX3*, *CYGB*, *APOE*, *DHCR24*, and *SEPP1*, following challenge with oxidative stress. We also measured *NQO1* expression by qPCR in untreated clones as described below. Gene expression of *P21* was detected to monitor cell cycle arrest after 8 h H_2_O_2_/BSO treatment. We employed validated primer sets for the specified genes (Qiagen, Hilden, Germany) in combination with the QuantiTect SYBR Green PCR Master Mix (Qiagen) and a basket housekeeper containing glyceraldehyde-3-phosphate dehydrogenase (*GAPDH*), ribosomal protein L13a (*RPL13A*), beta-2-microglobulin (*B2M*), and TATA box binding protein (*TBP*). The basket housekeeper was amplified and the mean cycle number at threshold (Ct) was used for comparative quantification. Expression levels were determined by an efficiency-corrected model and by using FH-hTERT control cells as calibrator (expression level = 1) as described [39].

### Statistical analysis

All experiments were performed with two to three repetitions. Data are presented as means ± standard deviation (SD). The unpaired Student's *t*-test was used for statistical analysis and P values less than 0.05 were considered statistically significant.

## SUPPLEMENTARY FIGURES


