# Supplementary material for: Constitutive gp130 activation rapidly accelerates the transformation of human hepatocytes via an impaired oxidative stress response
Source: Oncotarget. 2016 Jul 30;7(34):55639–48. doi: 10.18632/oncotarget.10956 (PMC5342442; doi:10.18632/oncotarget.10956)
Supplement: Supplementary file 1 [file oncotarget-07-55639-s001.pdf]

Constitutive gp130 activation rapidly accelerates the transformation of human hepatocytes via an impaired oxidative stress response

SUPPLEMENTARY FIGURES

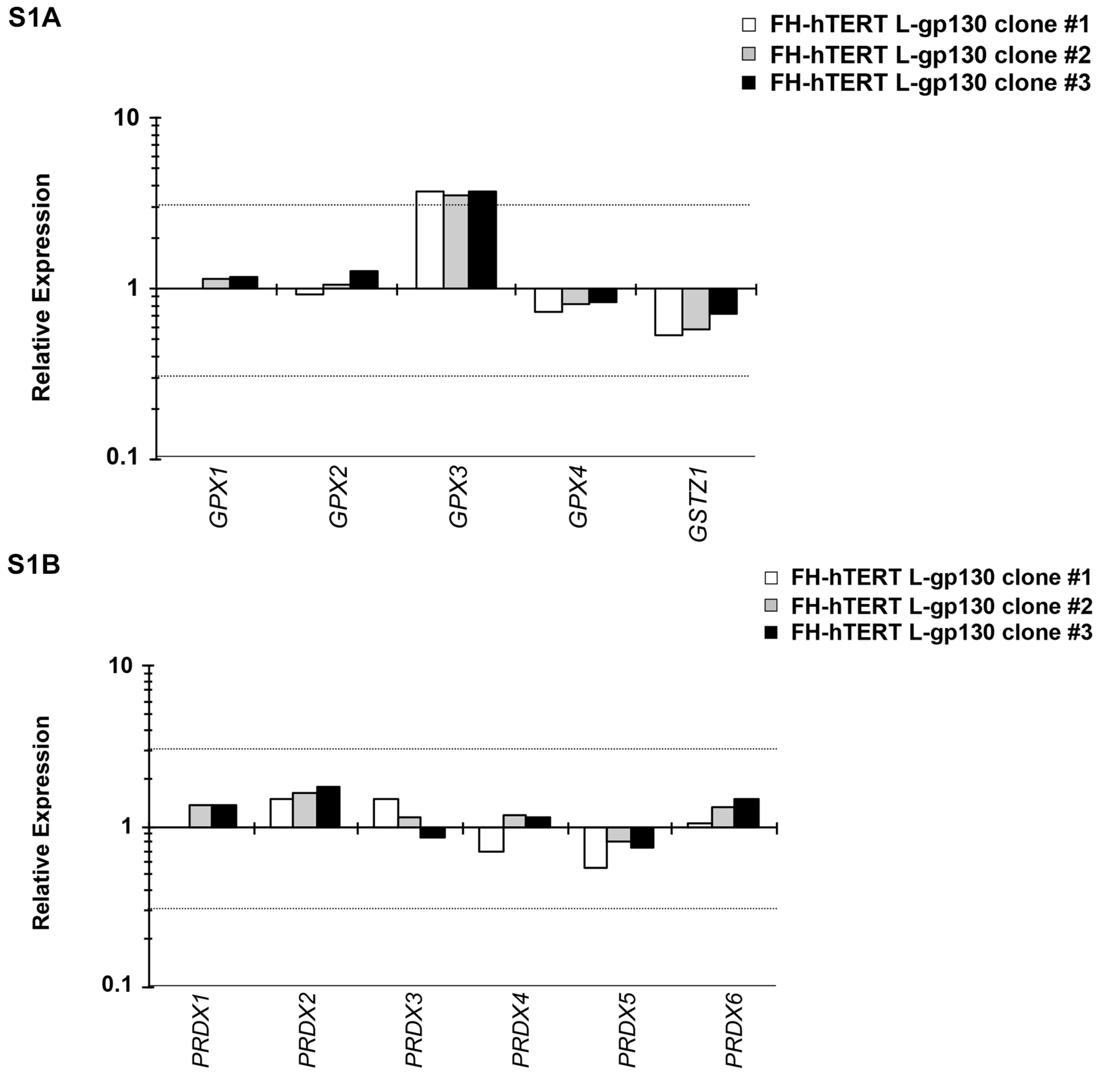

Supplementary Figure S1: Human Oxidative Stress and Antioxidant Defense RT² Profiler™ PCR Array to explore antioxidative stress response. Expression studies were conducted in mock-transfected FH-hTERT and the three L-gp130 clones employing qPCR array technology profiling genes involved in antioxidant defense. Normalized expression levels were compared with mock-transfected cells as calibrator (expression level = 1). The selected cutoff value (relative expression +/- factor 3) is depicted as dashed line. Assessed genes are related to oxidative stress: **A.** Glutathione peroxidases; **B.** peroxiredoxins; (*Continued*)

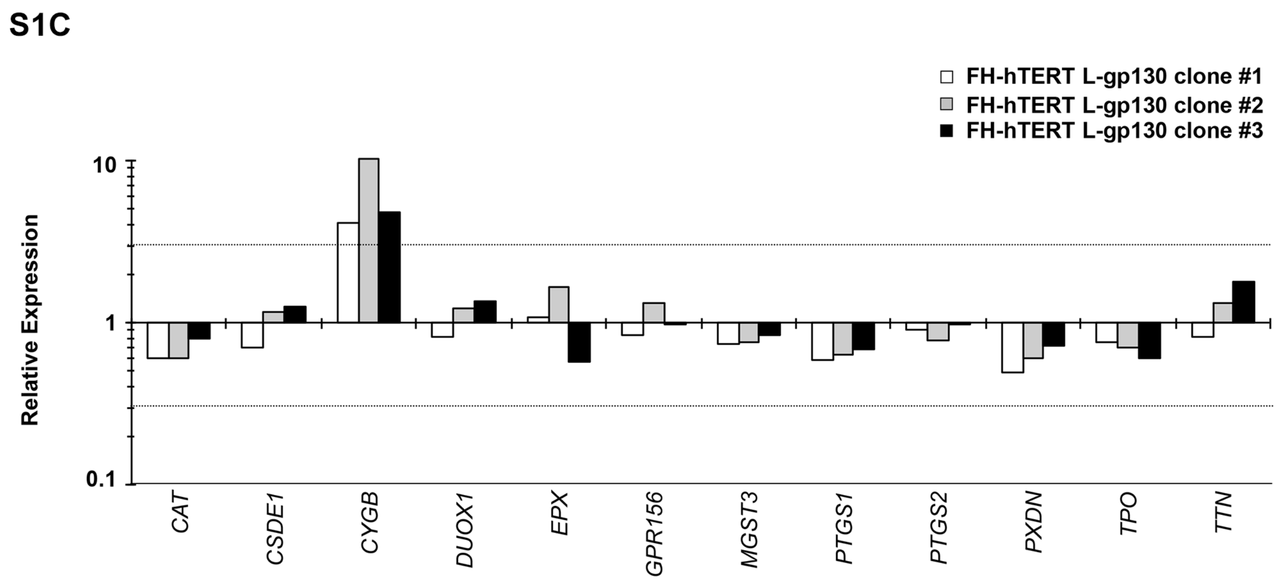

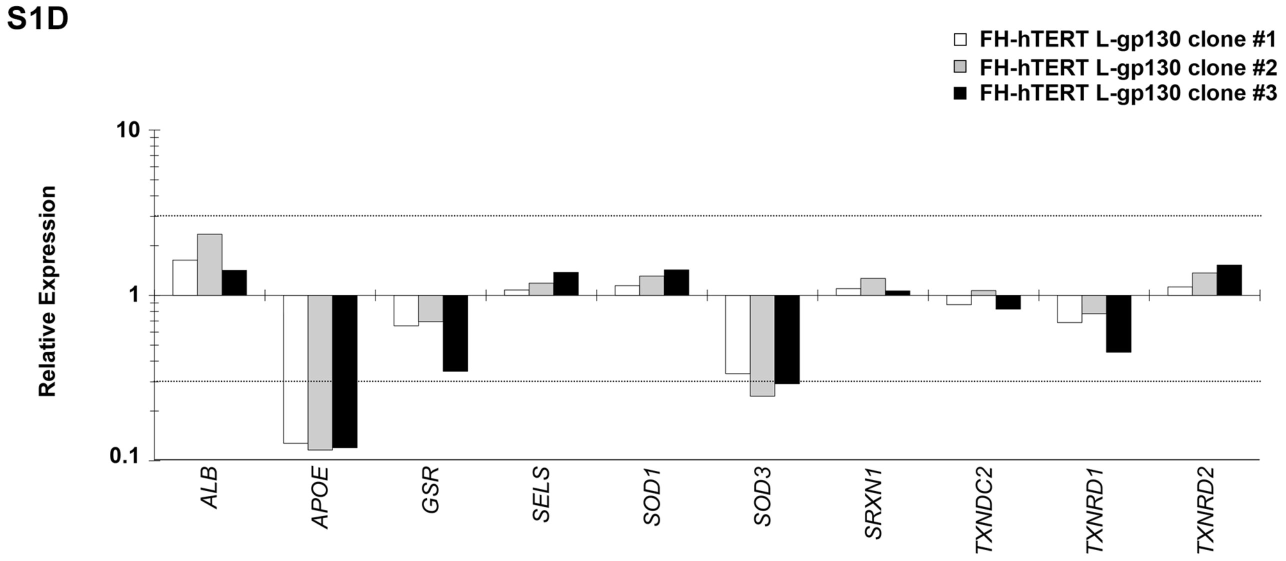

Supplementary Figure S1: Human Oxidative Stress and Antioxidant Defense RT² Profiler™ PCR Array to explore antioxidative stress response. C. other peroxidases; **D.** other antioxidants; (*Continued*)

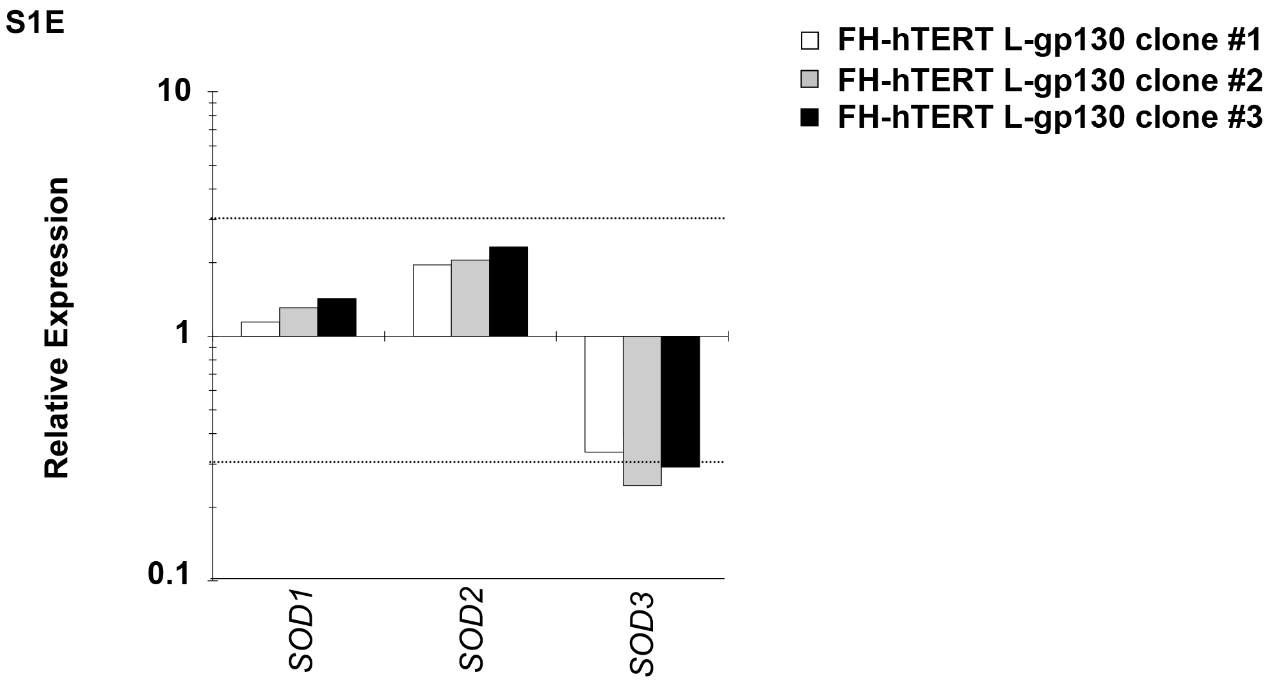

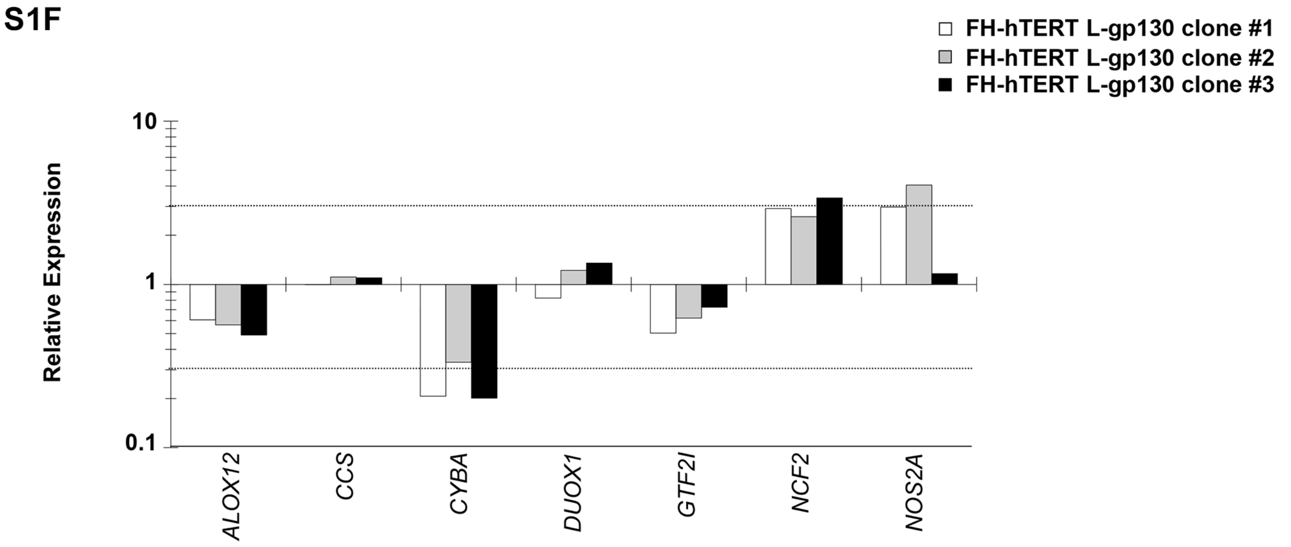

Supplementary Figure S1: Human Oxidative Stress and Antioxidant Defense RT² Profiler™ PCR Array to explore antioxidative stress response. E. superoxide dismutases; **F.** other genes involved in superoxide metabolism;

S1G

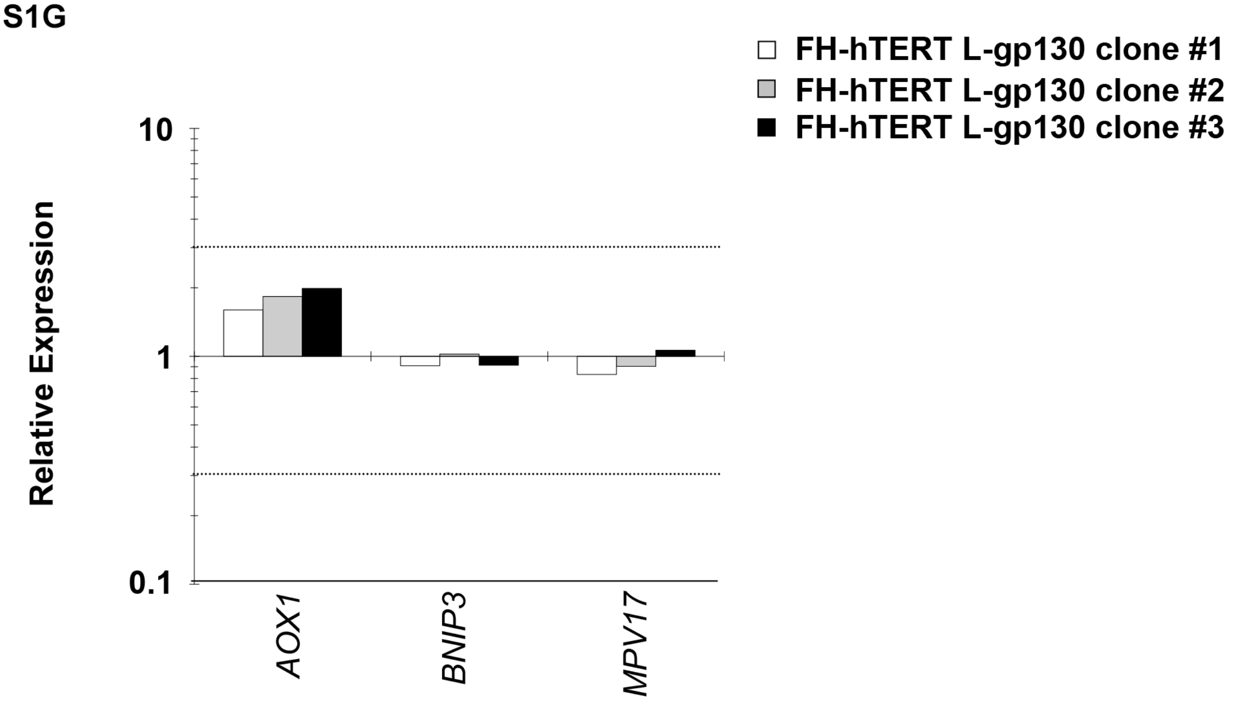

S1H

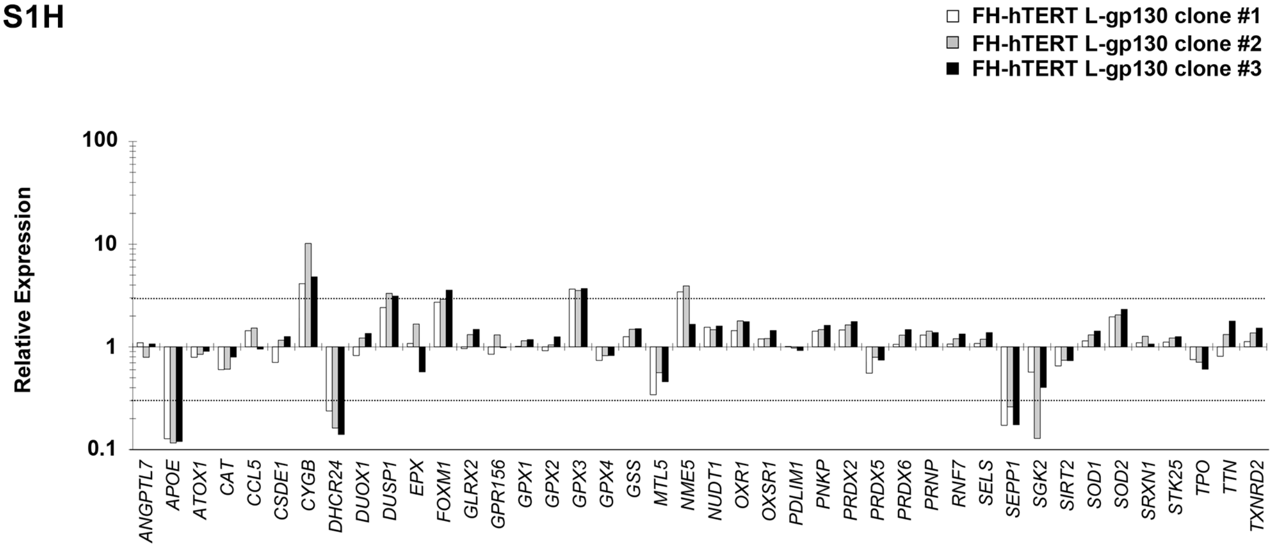

Supplementary Figure S1: Human Oxidative Stress and Antioxidant Defense RT² Profiler™ PCR Array to explore antioxidative stress response. G. other genes involved in ROS metabolism; and **H.** oxidative stress responsive genes.

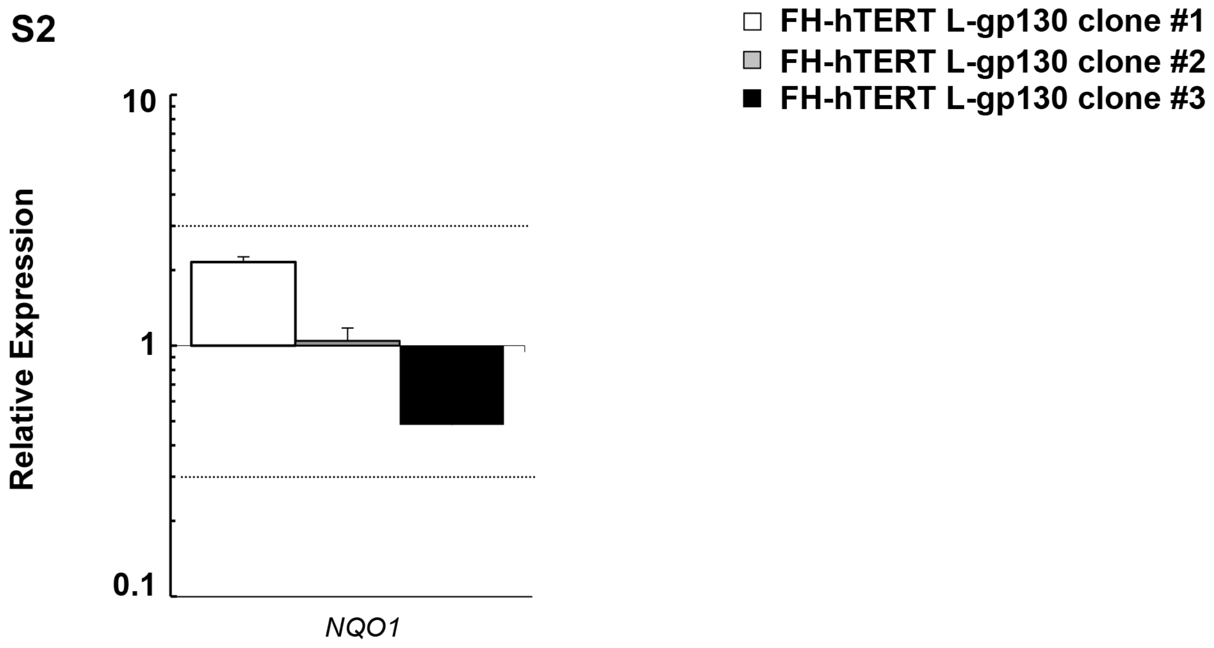

Supplementary Figure S2: Relative expression of cytoprotective NAD(P)H dehydrogenase quinone (NQO1) as major transcriptional target of Nrf2 in our gp130-activated clones ± SD (error bars). Normalized expression levels were compared with mock-transfected cells as calibrator (expression level = 1).
